# Global profiling and annotation of templated isomiRs dynamics across *Caenorhabditis elegans* development

**DOI:** 10.1080/15476286.2022.2099646

**Published:** 2022-07-18

**Authors:** Ganesh Panzade, Li Li, Shilpa Hebbar, Isana Veksler-Lublinsky, Anna Zinovyeva

**Affiliations:** a Division of Biology, Kansas State University, Manhattan, Kansas, USA; b Department of Software and Information Systems Engineering, Ben-Gurion University of the Negev, Beer-sheva, Israel

**Keywords:** microRNAs, miRNAs, isomiRs, *C. elegans*, development

## Abstract

microRNAs (miRNAs) are small non-coding RNAs that regulate gene expression through translational repression and mRNA destabilization. During canonical miRNA biogenesis, several miRNA isoforms, or isomiRs, are produced from a single precursor miRNA. Templated isomiRs are generated through Drosha or Dicer cleavage at alternate positions on either the primary or the precursor miRNAs, generating truncated or extended 5’ and/or 3’ miRNA ends. As changes to the mature miRNA sequence can alter miRNA gene target repertoire, we investigated the extent of templated isomiR prevalence, providing a profiling map for templated isomiRs across stages of *C. elegans* development. While most miRNA loci did not produce abundant templated isomiRs, a substantial number of miRNA loci produced isomiRs were just as, or more, abundant than their annotated canonical mature miRNAs. 3’ end miRNA alterations were more frequent than the seed-altering 5’ end extensions or truncations. However, we identified several miRNA loci that produced a considerable amount of isomiRs with 5’ end alterations, predicted to target new, distinct sets of genes. Overall, the presented annotation of templated isomiR dynamics across *C. elegans* developmental stages provides a basis for further studies into miRNA biogenesis and the intriguing potential of functional miRNA diversification through isomiR production.

## Introduction

MicroRNAs (miRNAs) are small non-coding RNAs that post-transcriptionally regulate gene expression and play a critical role during animal development. miRNAs base-pair with complementary sequences in 3’ UTRs of target messenger RNAs (mRNAs), effecting their translation repression and/or mRNA degradation [1]. Most mature miRNAs are generated through a canonical biogenesis pathway, which begins when miRNA genes are transcribed by RNA-Pol II to produce long primary miRNA transcripts (pri-miRNAs) [1,2]. Each pri-miRNA is then processed by a nuclear Drosha/DGCR8 ribonuclease enzyme complex to generate a precursor miRNA (pre-miRNA), characterized by a hairpin, or a stem-loop, secondary structure. In addition to canonical miRNA biogenesis, non-canonical biogenesis pathways have been described. miRtrons are miRNA loci embedded in introns that produce short hairpin precursor miRNAs generated by splicing and debranching rather than Drosha/DGCR8 cleavage [reviewed in 3, 4]. A subclass of miRtrons have 5’ or 3’ extended tails undergo additional end trimming to generate the short precursors [4]. The pre-miRNAs, generated by either the canonical or non-canonical pathways, are exported to the cytoplasm by Exportin-5 and further processed by the Dicer enzyme into a mature miRNA/miRNA* duplex with two nucleotide 3’ overhang [2], although exceptions do exist [5–7]. One of the strands of this duplex is loaded into an Argonaute protein (AGO) to form the core of the miRNA-induced silencing complex (miRISC). This complex interacts with miRNA complementary binding sites in 3’ UTRs of target mRNA, setting off a cascade of molecular events that lead to translation repression and/or mRNA degradation [1,2].

Mature miRNAs are usually annotated as a single sequence (https://www.mirbase.org/, https://mirgenedb.org/). Nevertheless, it was observed early on that miRNA loci frequently produce miRNA sequence variants of different lengths [8]. These variants, known as isomiRs, were initially considered experimental artefacts with limited biological relevance, and thus were discarded [9–11]. Initially, spike-in synthetic RNA oligonucleotides were used to demonstrate that isomiR rates exceeded the error rates of next-generation sequencing [12]. Further use of advanced next-generation sequencing and new computational tools [13–15] led to the identification of a vast array of isomiRs from various species in much higher frequencies than was previously appreciated [16,17]. Moreover, co-immunoprecipitation experiments demonstrated that isomiRs are loaded into Argonaute (AGO) and can interact with target mRNAs [18–21]. These lines of evidence strongly support the functional importance of observed isomiRs.

**Figure 1.**
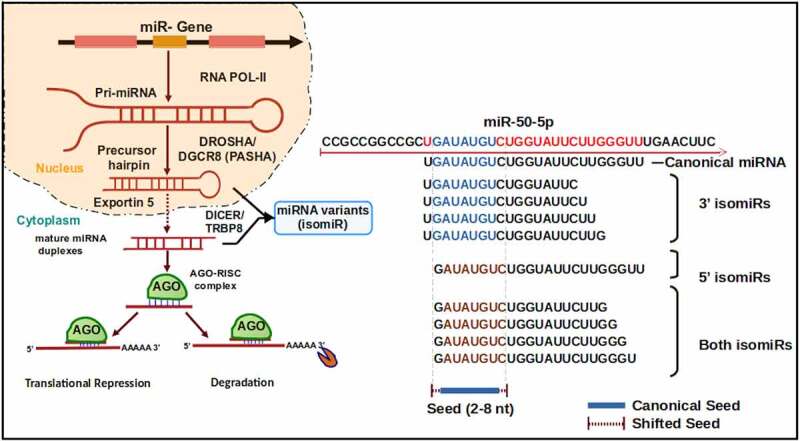
**Overview of miRNA biogenesis and isomiR production through alternative cleavage events**. miRNAs undergo two processing events resulting in the mature::star miRNA duplexes. First, Drosha processes the primary miRNA (pri-miRNA) into the hairpin precursor miRNA (pre-miRNA). Then, Dicer cleaves the loop of the pre-miRNA to generate the miRNA duplex. After Argonaute loading, one of the duplexed strands is ejected, leaving the mature miRNA strand loaded and ready to guide Argonaute to its target genes. Imprecise cleavage by Drosha and/or Dicer produces truncated or extended 5’ and/or 3’ miRNA ends, generating alternative miRNA isoforms, or isomiRs. Shown are examples of *miR-50-5p* isomiRs recovered from the *C. elegans* small RNA sequencing data, which may represent alternative cleavage isoforms produced during miRNA biogenesis process.

IsomiRs can be generated during miRNA biogenesis via alternative enzymatic processing of pri-miRNA and pre-miRNA precursors or through different post-miRNA maturation events (Fig. 1)[2,22]. During the canonical miRNA biogenesis pathway, the 5’ and 3’ ends of the miRNAs are determined by consecutive cleavage events of the pri-miRNA and pre-miRNA by the enzymes Drosha and Dicer [2]. Imprecise Drosha and/or Dicer cleavage generates miRNA molecules that match the reference genome sequence but show heterogeneity in length, known as templated isomiRs (Fig. 1) [9,23]. Non-canonically generated miRNA loci, such as miRtrons, can also produce isomiRs due to imprecise cleavage by Dicer and/or end trimming. Shorter templated miRNA sequences can also be produced by exonuclease nibbling activity at the ends of the miRNA [9]. Length and sequence heterogeneity that arises by the addition of one or more bases at the 3’ end of miRNAs generates ‘non-templated isomiRs’, where added nucleotides may not match the reference genome [9]. Non-templated nucleotide additions occur at the 3’-end of a miRNA, with the most prevalent additions being uridylation or adenylation [24]. Although less frequent, some isomiRs, known as polymorphic isomiRs, contain internal nucleotides different from the genomic sequence due to RNA editing events [22,25]. Overall, isomiRs can be categorized into four main classes based on variation in length and/or sequence: 5’ isomiRs with changes in length at the 5’ end, 3’ isomiRs with changes in length at the 3’ end, polymorphic isomiRs with identical length but with nucleotide changes within the mature sequence, and mixed type isomiRs with changes in length and sequence [9,15,22].

3’ isomiRs are the most common miRNA isoforms in animals and plants, both in terms of the number of miRNAs displaying these variations as well as their overall abundance [24,26]. Though these isomiRs have the same seed sequence as the canonical miRNA, non-templated 3’ end nucleotide additions have been reported to affect miRNA loading into the miRISC, miRNA stability, and miRNA targeting characteristics [18, 27; 17, 28, 28, 29; 30]. For example, upregulation of a longer, templated 3’ isomiR of miR-222 promotes apoptosis by inhibiting genes in the PI3K–AKT pathway such as PIK3R3, opposing the anti-apoptotic role of the canonical miR-222 [31].

While 5’ isomiRs are rare, they still represent a significant proportion of the total sequence population of some specific miRNAs [9,32]. 5’ isomiRs are functionally important as they shift the 2–8nt miRNA seed sequence and likely affect the miRNA target repertoire [32]. Several studies have reported that the 5’ isomiRs expand the functional target gene pool of their miRNAs [33–35]. A 5’ isoform of the neuronal-specific miR-124a-3p gains a new target, CDH11, a gene involved in retinal differentiation that is not typically regulated by the canonical miR-124a-3p [36]. A 5’ isomiR of miR-411 was found to be abundantly expressed in human primary vascular cells, with 5’ miR-411 isomiR negatively influencing vascular cell migration [37].

Several studies reported variable isomiR expression patterns across different tissues or cell lines [21,32,38,39]. Distinct patterns of isomiR expression suggest that isomiR production could be regulated in a tissue or developmental stage-specific manner, with expression dynamics varying in response to biological stimuli [24,26]. miRNA sequencing from human embryonic, neural, and foetal mesenchymal stem cells similarly showed unique proportions of isomiRs among cell and tissue types [32]. Recent isomiR expression analysis from single cell small RNAseq data across cell lines also demonstrated unique isomiR profiles across distinct cell types [40]. A comprehensive study of isomiR expression across 32 normal and tumour tissues from The Cancer Genome Atlas (TCGA) demonstrated that differentially expressed isomiRs could discriminate between the 32 cancer types [39]. A recent study in *C. elegans* profiled miRNAs loaded into silencing complexes across three major tissue types (intestine, body wall muscles, and nervous system), identifying a rich array of miRNA isoforms that exhibited cell- and AGO-specific loading patterns [41]. However, a detailed profile of *C. elegans* templated isomiRs across development remains unknown. As alternative processing can affect miRNA strand selection and loading into Argonauts [42], a detailed analysis of templated isomiRs forms a critical baseline for studies aimed at understanding regulation of Argonaute programming with alternative miRNA strands.

In this study, we performed a comprehensive analysis of the canonical miRNAs and their templated isomiRs across *C. elegans* developmental stages. Specifically, we used a computational pipeline to investigate the dynamic patterns of miRNA-locus derived canonical miRNA sequences and their templated isomiR populations. We found variable isomiR patterns for miRNA loci, ranging from loci that produce only a small amount of isomiRs to loci which produced abundant isomiR species. In some cases, isomiR prevalence changed across the developmental stages, suggesting that developmental functional specificity of isomiRs may exist. Not surprisingly, 3’ end miRNA alterations were more frequent than the potentially seed-altering 5’ end extensions or truncations. Nevertheless, we identified several miRNA loci with abundant 5’ isomiRs predicted to target new, potentially distinct sets of genes. Overall, we provide miRNA and templated isomiR dynamic maps across *C. elegans* developmental stages that could provide insights into miRNA biogenesis and the intriguing potential of developmentally-regulated isomiR function.

## Materials and methods

### RNA preparation and small RNA sequencing

Wild type *C. elegans* animals (N2) were cultured under standard growth conditions on NGM plates using OP50 as a food source [43]. Animals were synchronized using bleaching [recipe #2, 44] and were collected when ~80% of animals were within the required stage (Embryo, L1, L2, L3, L4, young adult (YA), and dauer). RNA preparation was performed as previously described [45]. Small RNAs were size selected by gel purification as described [46]. Small RNA libraries were prepared from the size-selected RNA using the NEXTflex Small RNA Library Prep kit v3 (Bioo Scientific) and sequenced on the Illumina NextSeq instrument at the Kansas State University Genomic Core. Each stage was represented by three biological replicates, except for embryos, which was represented by four replicates (Supplemental Table 1). A sequencing dataset for the L4 stage was retrieved from [47].

### Data analysis

We first assessed the quality of the raw sequencing reads with FastQC v0.11.8 (https://www.bioinformatics.babraham.ac.uk/projects/fastqc/). The data was then processed using cutadapt tool [48] to retain reads with a quality score (QS) above 25% and to clip 5’ and 3’ adapters. Clipping and further read processing was performed as follows. The adapter sequence was clipped from the 3’ end (*-a ATCTCGTATGCCGTCTTCTGCTTG -e 0.1*). Reads were split to barcoded libraries using fastx_barcode splitter utility (http://hannonlab.cshl.edu/fastx_toolkit/index.html) based on respective barcode index sequences. Files that belonged to the same barcode were concatenated; the remaining 3’ end and 5’ adapter sequences were clipped *(-a* TGGAATTCTCGGGTGCCAAGGAACTCCAGTCAC -g

AATGATACGGCGACCACCGAGATCTACACGTTCAGAGTTCTACAGTCCGA -e 0.1). Reads containing 4nt randomers on both sides were mapped to reference genome index by subread aligner [49] with following parameters (-n 35 – m 4 – M 0 – T 20 – I 0 – B 1). We extracted alignments with CIGAR flag of the form ‘4S*M4S’ (indicating that the first and last four nucleotides were soft-clipped) into sorted BAM file for further trimming. The first and last 4 randomer bases were trimmed, and reads with a final length ranging between 17–29 nt were selected for further analysis.

**Figure 2.**
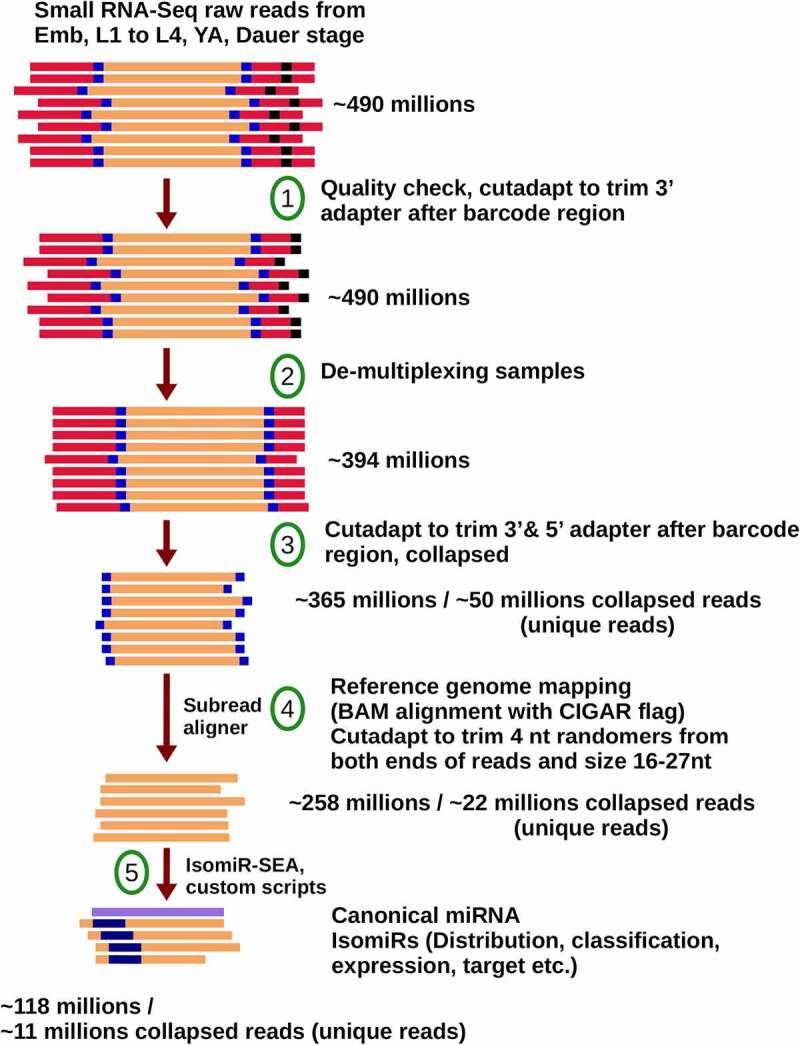
A custom pipeline developed to identify *C. elegans* isomiRs from filtered and collapsed reads generated by small RNA sequencing (for details, see Supplemental Table 1).

A custom script was used to collapse all identical reads for unique tag representation while retaining their respective counts into a FASTA file. The FASTA file was then converted into a tab-delimited file containing the read sequence tags and their corresponding counts to serve as input for isomiR identification by isomiR-SEAv1.6 (Fig. 2, 14). In addition, correlation analysis was performed on reads from small RNAseq replicates to ensure consistency among the biological replicates (Supplemental Figure 1).

### IsomiR identification

We utilized the standalone tool isomiR-SEAv1.6 [14] for isomiR identification. Briefly, IsomiR-SEAv1.6 performs miRNA seed search of miRNAs that are provided as a reference in the input tag sequences. Once a perfect seed match is found in the tag sequence, the alignment between the miRNA and the tag is extended in both directions to cover the offset and supplemental sequence region. The positions of the encountered mismatches are recorded and analysed to distinguish among miRNAs and isomiRs. We ran IsomiR-SEAv1.6 with a species code ‘cel’, a file containing reference mature miRNAs (downloaded from miRBase version 22) [50], and a directory containing the tab-delimited files with unique tags and their counts, with each file corresponding to a sample, generated as explained above. The rest of the parameters included a minimum tag length (-l 16), seed size (-ss 6), and tag selection threshold (-h 11).

### Post-processing isomiR-SEA results

We processed the isomiR-SEA output files to retrieve read counts for the annotated canonical miRNAs and their templated isomiRs across all stages. The raw read counts were normalized to reads per million (RPM) of total miRNA counts in each library. We retrieved the isomiR template information from the output files across all stages using a custom perl script and grouped them according to their end variations (5’, 3’, or both). We then annotated the isomiRs according to the type of variation, addition (extension) or deletion (truncation), and the number of added and/or deleted bases compared to their canonical miRNAs.

### Distribution of isomiRs and contribution of Drosha/Dicer processing to isomiRs pool

We collected all isomiRs according to their class type (5’, 3’, or both) and computed the number of unique tags and their total read abundance for each isomiR type in each stage. The percentage within each class was calculated by dividing the unique tags or read abundance value by the total unique tags or total read abundance, respectively. Next, we aligned all isomiR sequences to miRNA precursor to identify the arm of origin (5p or 3p) using Clustal [51]. We categorized the isomiR sequences that aligned to the 5p arm as 5’ DROSHA or 3’ DICER if the sequence variation occurred at the 5’ or 3’ end of the 5p isomiR, respectively. IsomiRs that had variation at both ends were labelled with both a 5’ and a 3’ tag. Similarly, we labelled the isomiRs that aligned to the 3p arm as 3’ DROSHA (if 3’ end was affected), or 5’ DICER (if 5’ end was affected), or both. For each of the four groups, we calculated the unique tag number and read abundance counts across all stages. All the above analysis was performed using custom scripts (https://github.com/gppbioinfo/isomiR_celegans).

### miRNA/isomiR end nucleotide variation

To compare the distribution of the first nucleotide of isomiRs to canonical miRNAs, we classified the sequences according to their first base (A, U, C, G). For each of the four bases, we compiled unique tag counts as well as read abundance counts for each stage. We calculated the percent prevalence for each base by dividing the unique tags or read abundance by the total unique tags or total read abundance, respectively. We performed a similar analysis for several types of end-variations (5’/3’, extension/deletion, number of bases).

### Data visualization

All plots were generated in R using the ggplot2 library (https://ggplot2.tidyverse.org). IsomiR sequences were sorted according to their expression across the stages and isomiR abundance was plotted for top six most abundant isomiRs. Multiple plots were combined by employing Montage Linux command in ubuntu and the figures were arranged using GIMP (https://www.gimp.org/) tools in ubuntu. Multiple sequence alignments of canonical miRNAs and their respective isomiRs were generated and visualized using JalView tool [52].

### IsomiR target information

Seed sequences, found at positions 2–8, were extracted for miRNAs and their corresponding isomiRs and formatted into a tab-delimited file (miR/isomiR id, seed sequence, and taxonomic species code from NCBI). IsomiR sequences that shared the same seed region were grouped into one seed cluster (i.e. 6239). *C. elegans* 3’ UTR sequences were downloaded from ENSEMBL Biomart and formatted into a tab-delimited file (geneid/symbol, species code, and UTR sequence). We supplied both files as input for TargetScanWorm6.2 standalone tool [53], which predicts miRNA:target gene interactions based on 8/7/6mer complementary to miRNA seed region. We collected all the target genes from miRNA:target gene interactions for all sites and used them for distinct set analysis between isomiRs and canonical miRNAs. We summarized the results with Venn diagrams using Venn (v0.1.3), a python3 module.

### Clustering of miRNAs

The normalized expression matrix of miRNAs and their isomiRs were utilized to plot a meta heatmap. The low count isomiRs were filtered out if their row-wise sum was less than 5 RPM. The plots were generated with pheatmap, an R package for interactive heatmaps (https://cran.r-project.org/web/packages/pheatmap). The matrix values were scaled across rows; row-wise clustering was done with the ‘average’ method.

## Results

To characterize the *C. elegans* templated isomiRs and to assess their dynamics across *C. elegans* development, we sequenced and analysed templated isomiR populations from embryos, L1, L2, L3, L4 larvae, young adults (YA), and dauer. To minimize bias introduced during the library preparation, we used a method that includes random sequences in both linkers to reduce linker ligation bias (see Materials and Methods). In addition, the method used to prepare the libraries includes PEG in the ligation buffers, which has been shown to further minimize bias [54]. Approximately 490 million raw reads were filtered, trimmed, and collapsed into 50 million unique tags while retaining count information (Fig. 2, Supplemental Table 1). To eliminate the additional complexity of untemplated additions and internal read polymorphisms we focused our analysis on miRNA reads that matched perfectly to the genome. Eleven million unique tags perfectly matched the genome and were subsequently used for templated isomiR identification (Fig. 2, Supplemental Table 1). Our analysis of the resulting data provides a detailed assessment of templated isomiR distribution across *C. elegans* development.

### miRNAs have dynamic expression across developmental stages

**Figure 3.**
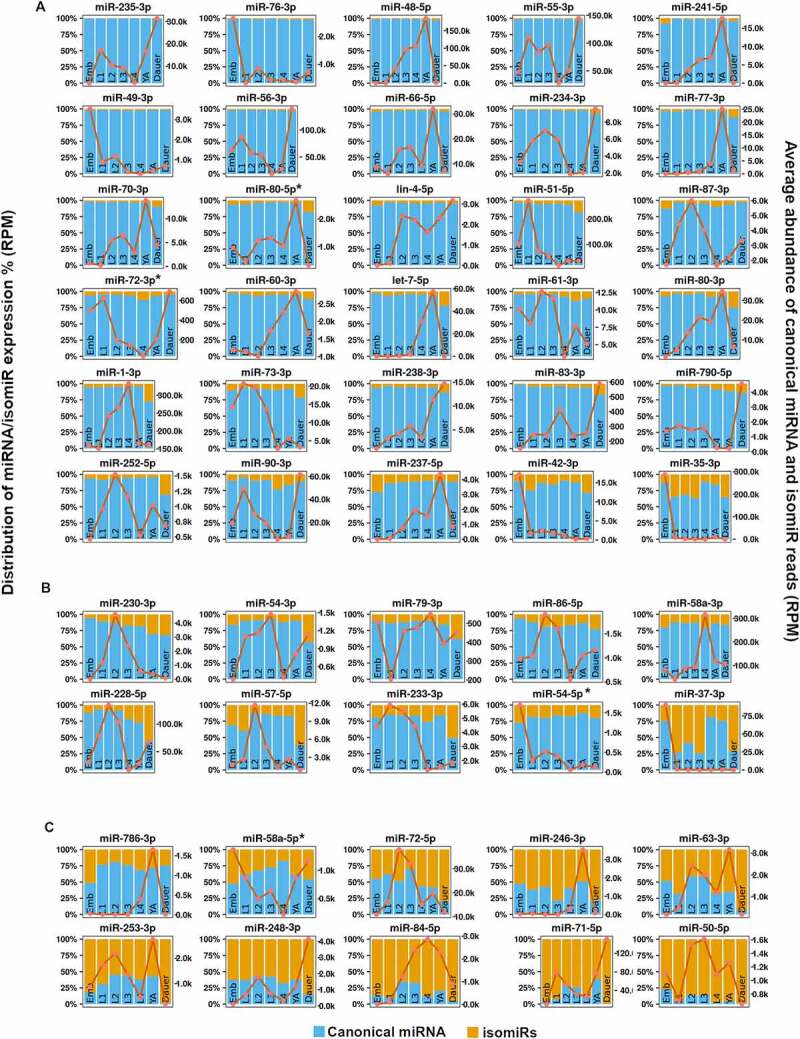
**Global profiling of *C. elegans* miRNAs and their isomiRs across developmental stages**. miRBase-reported *C. elegans* miRNAs were scanned for templated variations at the 3’ and 5’ ends. Distribution of reads mapping to miRNA loci (left y-axis), with canonical reads, identical to miRBase-reported major miRNA species, are displayed in blue and isomiR reads are shown in yellow. Orange line denotes the total abundance of miRNA loci-mapped reads, including canonical miRNAs and their isomiRs (reads per million, right y-axis). Shown are the fifty most abundant miRNAs grouped into panels A-C based on the proportion of canonical miRNA reads out of total miRNA and isomiR reads summed across all stages: (A) ≥ 85%; (B) <85% and ≥70% and (C) <70%. * denotes miRNA passenger or miR* strands. For remaining miRNA/isomir expression dynamics, see Supplemental Figure 1.

To fully characterize templated isomiR populations and their dynamics across *C.* *elegans* developmental stages, we first examined our data for overall miRNA abundances. To broadly ensure the quality of our data and examine overall miRNA dynamics across stages, we plotted the average abundance of all reads perfectly mapped to miRNA loci, including canonical miRNAs and their isomiRs (Fig. 3, top 50 most abundant miRNAs, and Supplemental Fig. 2, remaining miRNAs detected in our analysis, and Supplemental File 1). We found that our data broadly agreed with previously reported miRNA profiling across *C. elegans* development [55–57]. Our miRNA abundance representation allows for quick determination of specific patterns across development, such as highest expression at certain stages (red line, Fig. 3 and Supplemental Figure S2). For example, several miRNAs, miR-87-3p (Fig. 3(A)), miR-86-5p and miR-228-5p (Fig. 3(B)), miR-248-3p (Fig. 3(C)), and miR-793 and miR-245-3p (Supplemental Figure S2), showed highest abundance at L2 and dauer stages, suggesting possible common roles in development and dauer formation and/or maintenance (Fig. 3, Supplemental Figure S2).

### Individual miRNA loci produce varying quantities of templated isomiR species

We found that for many miRNA loci, the most abundant miRNA isoform present in our data was the canonical miRNA, the isoform previously annotated as mature miRNA by miRbase (Fig. 3(A), Supplemental Figure S2, Supplemental File 1). Templated isomiRs made up less than 15% of total reads for a large number of miRNAs, including heterochronic miRNAs miR-241-5p, let-7-5p, and lin-4-5p (Fig. 3(A), Supplemental Figure S2). Other miRNA loci generated miRNA isoforms that primarily consisted of the canonical miRNA species, yet on average showed a significant proportion of reads representing miRNA isomiRs (15–30%) (Fig. 3(B), Supplemental Figure S2). For example, the highly abundant miR-58a-3p and miR-228-5p exhibited such distribution (Fig. 3(B)). Other miRNA loci produced isomiR species that were, on average, higher in abundance than the previously annotated canonical miRNAs (Fig. 3(C), Supplemental Figure S2). This last category may represent previously mis-annotated miRNAs, possibly due to differences in library preparation methods. As less biased methods are used for small RNA library preparation in the future it would be important to continue to refine the exact sequence of the most abundant miRNA species. This is especially critical for any of the isoforms that include alterations to miRNA seed-changing 5’ end. However, as 3’ end changes have been suggested to alter miRNA-target interactions and potentially shift the target repertoire [31,58,59], accurate refinement of 3’ miRNA termini annotations may be similarly important.

### Developmental dynamics of miRNA levels vary among family members

**Figure 4.**
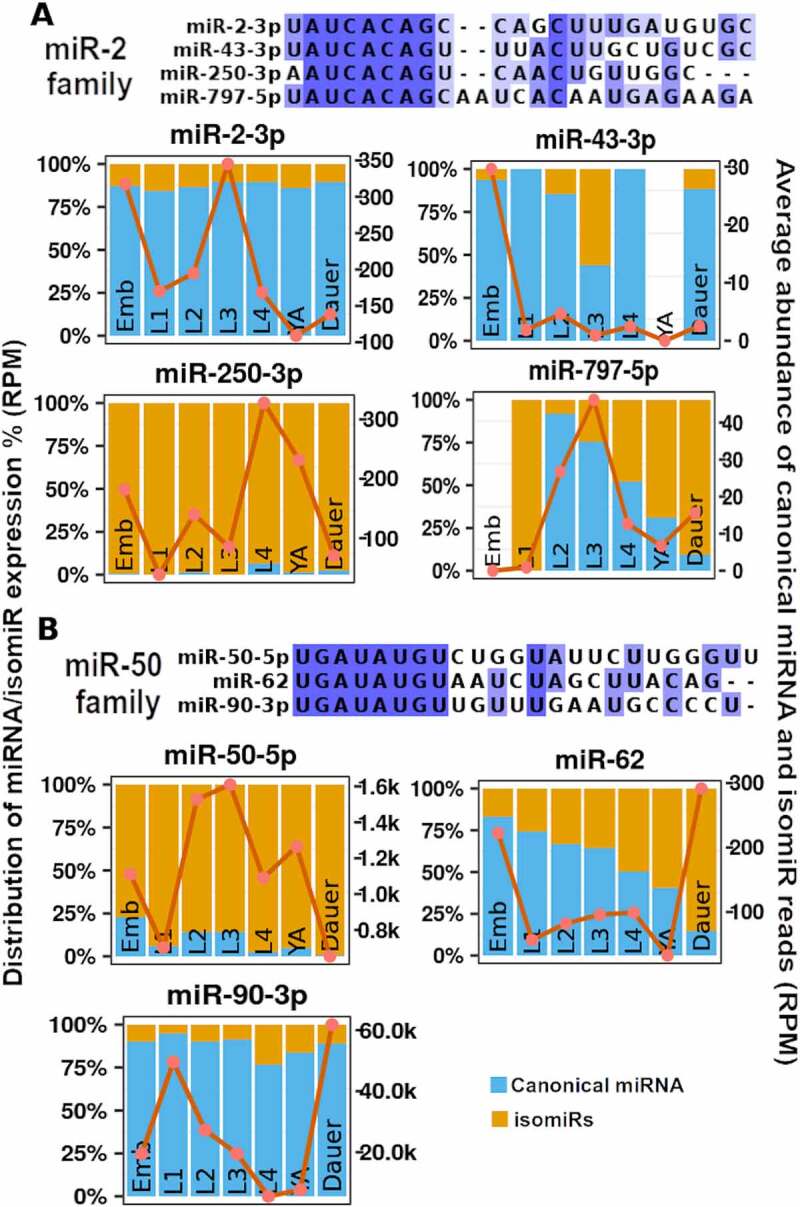
**miRNA and isomiR developmental expression dynamics vary among miRNA family members**. Sequence alignments of miRNAs belonging to the same family are provided with the temporal dynamics across stages. (A) Members of the *mir-2* family, miR-2-3p, miR-43-3p, miR-250-3p, and miR-797-5p and (B) members of the *mir-50* family, miR-50-5p, miR-62, and miR-90-3p display distinct temporal dynamics in total miRNA expression as well as canonical miRNA/isomiRs distributions across *C. elegans* stages. For remaining miRNA family expression dynamics, see Supplemental Figure 3. miRNAs whose levels were not detected in our analysis (possibly due to perfect genome mapping requirement) are not shown.

The dynamic temporal patterns of miRNA abundances across development prompted us to ask whether miRNAs belonging to the same miRNA family displayed similar patterns of expression across *C. elegans* developmental stages. Members of some families, such as *mir-36* family, unsurprisingly displayed identical patterns of expression (high in embryo, low or absent at other stages) (Supplemental Figure 3A). In contrast, members of other miRNA families showed distinct expression dynamics across the stages (Fig. 4, Supplemental Figure S3). For example, members of the miR-2 family, miR-2-3p, miR43-3p, miR-250-3p, and miR-797-5p, exhibited both overlapping and distinct temporal patterns (Fig. 4(A)). Both miR-2-3p and miR-797-5p reached peak abundance during the third larval (L3) stage while family members miR-43-3p and miR-250-3p remained low during the L3 stage, peaking instead during embryonic development and the fourth larval (L4) stage, respectively (Fig. 4(A)). miR-50 family miRNAs also displayed distinct temporal expression patterns, with miR-50-5p being most abundant during larval development and in young adults (Fig. 4(B)). miR-62 and miR-90-3p reached highest abundance in dauers (Fig. 4(B)). Despite single-family miRNAs’ potential to target the same genes due to shared seed sequence, temporal separation of miRNA expression may suggest distinct biological targets and functions. The observed temporal dynamics, combined with potential distinct spatial expression can further increase the functional separation of same-family miRNAs. As future studies identify *bona fide* targets of individual miRNAs, it will be interesting to see the true extent of target/functional overlap among family members.

### Majority of templated isomiRs display altered 3’ ends

**Figure 5.**
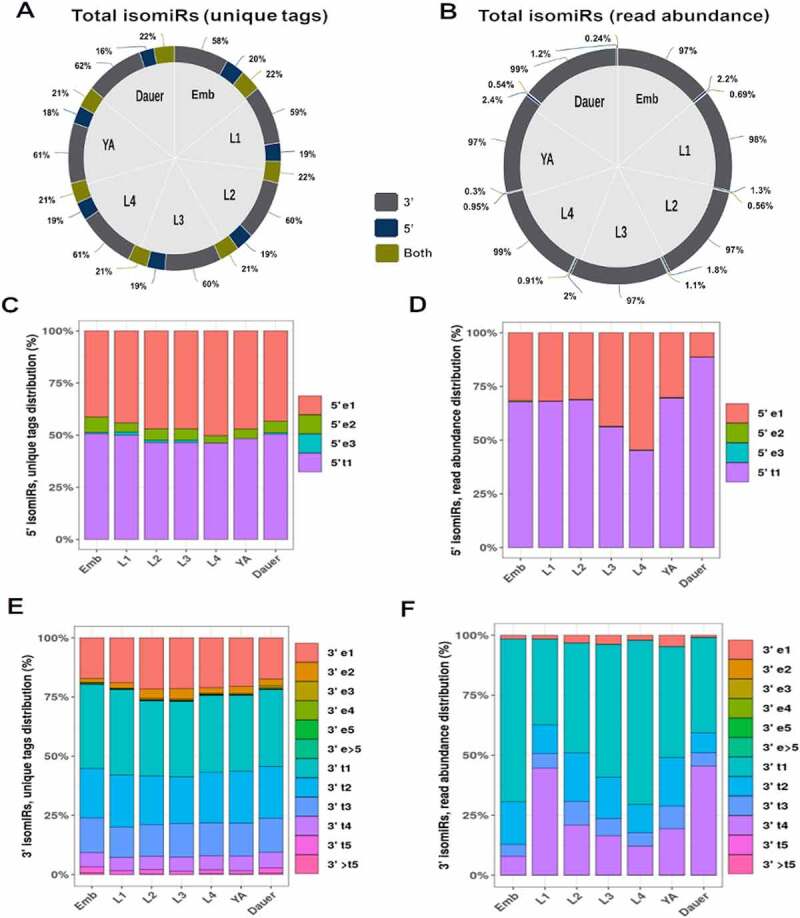
**IsomiR terminal sequence variability**. Majority of isomiRs exhibit alternative 3’ terminus ends both at the level of unique tag count (A) and overall abundance (B). Most of the 5’ alterations are limited to single nucleotide extensions and truncations when unique tags are considered (C), with single nucleotide truncations accounting for majority of the total isomiR reads by abundance (D). 3’ isomiRs exhibit a greater level of terminal diversity both when unique tags (E) and overall abundance (F) are examined, with a strong bias for truncations. Note that 3’ truncations could be alternative processing isoforms, products of degradation, or a combination of both.

miRNA gene targeting is primarily dictated by its seed region (nucleotides 2–8) [74] with supplemental 3’ end binding contributing to gene target selection [58,60]. Therefore, 5’ end alterations of miRNAs would have significant effects on target gene populations as 5’ truncations or extensions alter the miRNA seed. Our analysis of the relative prevalence of 5’ vs 3’ templated isomiRs showed that 3’ end extensions or truncations predominated in both unique tag count (Fig. 5(A)) and overall abundance (Fig. 5(B)), (Supplemental File 1). 5’ end alterations were present in up to 20% of unique isomiR sequence tags (Fig. 5(A)) but accounted for less than 3% of all isomiR reads by abundance (Fig. 5(B)). IsomiRs with altered 5’ and 3’ ends represented 21–22% of unique isomiR reads, but on average less than 1% of isomiR populations by read abundance (Fig. 5(A,B)).

We next wanted to determine the extent to which 5’ and 3’ miRNA ends were altered (Fig. 5(C-F), Supplemental File 2). We found that, on average, (~48%) of the 5’ isomiRs had a one nucleotide truncation, while ~46% of 5’ isomiRs had a single nucleotide templated extension when unique tag sequences were considered (Fig. 5(C)). By abundance, an average of 66% of 5’ isomiR reads had a single nucleotide truncation (Fig. 5(D)). The proportions of 5’ end alterations were similar across all stages, with the exception of dauers (Fig. 5(D)), suggesting that under normal development equal constraints may govern generation of these isomiRs.

Not surprisingly, a much larger variety of 3’ modifications were observed. One nucleotide truncations (33%, on average), two nucleotide truncations (21% across stages), and one nucleotide extensions (20%) accounted for majority of unique isomiR sequences (Fig. 5(E)). Single nucleotide truncations made up most of 3’ isomiR reads by abundance (35–68%, Fig. 5(F)). Interestingly, isomiRs truncated by 4nt at the 3’ end were a sizable proportion of reads by abundance, perhaps representing miRNA molecules undergoing degradation rather than functional isomiRs (Fig. 5(F)).

### DROSHA and DICER both contribute to 5’ and 3’ isomiR generation, with 5’ isomiRs displaying broad nucleotide signatures compared to canonical miRNAs

**Figure 6.**
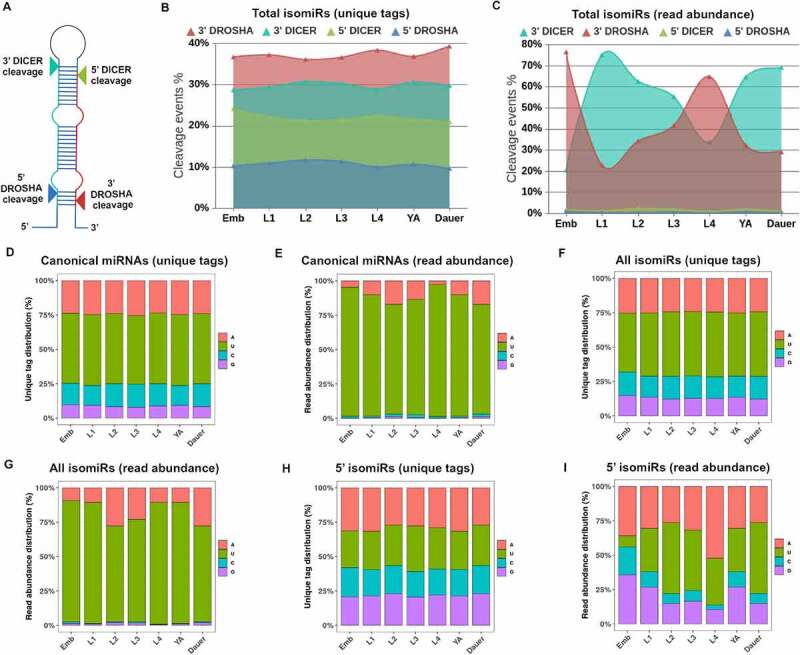
**Templated isomiRs can be produced by both Drosha and Dicer and show mild to moderate shifts in 5’ nucleotide identities**. (A) Canonical cleavage sites of Drosha and Dicer on the pri-miRNA and pre-miRNA, respectively, are indicated with arrows. Imprecise cleavage by these enzymes to either side results in either truncated or extended miRNA species. (B-C) Alternative cleavage by Drosha and Dicer of the 5’ and 3’ ends of the 5p/3p miRNA is denoted as 5’ Drosha, 3’ Drosha, 5’ Dicer, and 3’ Dicer, where 3’ end truncated isomiRs could represent both alternate cleavage products and miRNA degradation intermediates. Nonetheless, (B) Dicer generates slightly higher numbers of unique isomiR tags (unique isomiR sequences), with contributions of Drosha and Dicer to unique isomiR sequence production remaining fairly unchanged across stages. (C) In terms of overall isomiR abundance, 3’ Dicer-generated isomiRs are more prevalent across all stages, with the exception of the embryonic and L4 stages, where 3’ Drosha-generated isomiRs are more prevalent. (D-I) 5’ nucleotide composition of canonical miRNAs (D, E), their 5’ and 3’ isomiRs (F, G), and seed changing 5’ isomiRs only (H,I). Both isomiR unique tags (**D,F,H**) and overall abundance (**E,G,I**) are shown for all *C. elegans* stages.

Templated isomiRs can be generated by DROSHA and DICER (DRSH-1 and DCR-1 in *C. elegans*) during miRNA biogenesis (Fig. 1), (Fig. 6(A)). Both 5’ and 3’ end miRNA truncations or extensions can be produced by either enzyme, depending on whether the miRNA originates from the 5p or the 3p arm of the precursor (Fig. 6(A)). To determine whether isomiR production depended more on DICER or DROSHA processing, we analysed the fraction of 5’ and 3’ isomiRs produced by either enzyme (Supplemental File 2). We found that there were no significant differences in the number of alternative cleavage events produced by DROSHA (on average, 48% across stages) vs. DICER (on average, 52% across stages) when considering unique isomiR tags (Fig. 6(B)). When isomiR abundance was considered, DICER produced more cleavage events than DROSHA at all stages except Embryo and L4, with 3’ end cleavages vastly more abundant than 5’ end cleavages (Fig. 6(C)).

Processing alterations that generate isomiRs can result in different 5’ and 3’ end nucleotides. Since miRNA end nucleotide identity is an important determinant for which miRNA strand out of the duplex is loaded into Argonaute, alternative miRNA ends may affect this strand selection process [61]. To determine whether *C. elegans* isomiR populations had an altered 5’ nucleotide composition, we analysed the reads mapping to both the canonical miRNAs (Fig. 6(D,E)), all isomiRs (Fig. 6(F,G)), and 5’ isomiRs (Fig. 6(H,I)). When all isomiR populations were considered, there were no significant changes in 5’ nucleotide composition compared to canonical miRNAs (Fig. 6(D-G)), likely due to the fact that the majority of isomiRs observed are 3’ end altering isomiRs (Fig. 5). Unsurprisingly, 5’ isomiRs showed a shift in 5’ nucleotide identity both when unique tags (Fig. 6(H)) and read abundance (Fig. 6(I)) were considered. Uracil (U) was found at the start of 5’ isomiRs at an average of 20% of unique tags (Fig. 6(H)) and 33% of all isomiRs by abundance across stages (Fig. 6(I)). Adenosine (A) was the most abundant 5’ nt of 5’ isomiRs in both unique sequences (average of 29%, Fig. 6(H)) and by read abundance (average of 36% across stages, Fig. 6(I)). This observation held true across all stages except for embryos (Fig. 6(I)). As 5’ isomiRs did not represent a significant proportion of total miRNA reads, they may be unlikely to play a significant role in miRNA target repression, with a few specific exceptions discussed below.

### Some canonical miRNAs and their isomiR species exhibit distinct dynamics across c. elegans development

**Figure 7.**
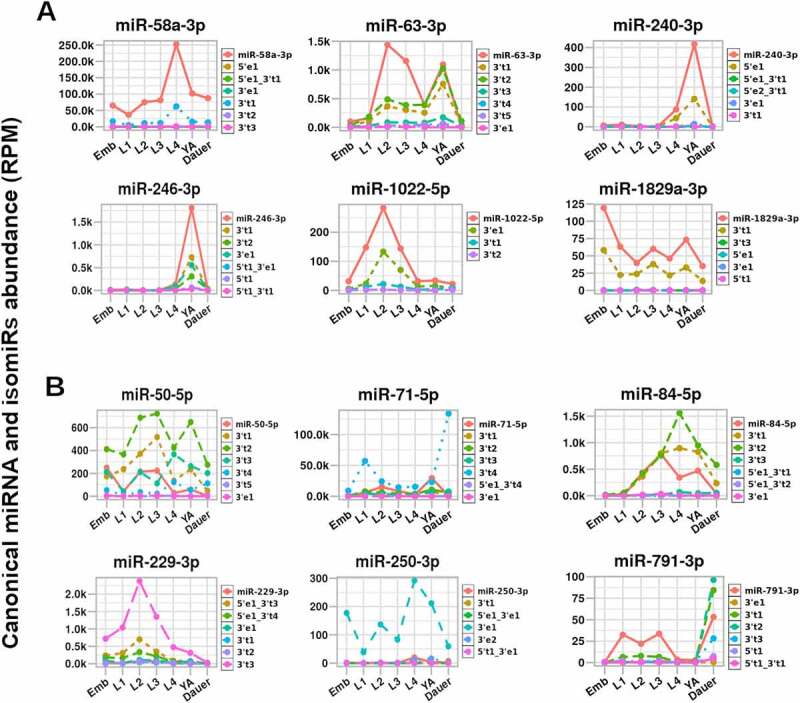
**Individual isomiR dynamics across *C. elegans* stages**. For some miRNAs, isomiRs exhibit unique dynamics, with specific isomiRs becoming more abundant than their canonical miRNA isoforms. (A) Examples of miRNAs whose isomiRs follow the developmental dynamics of their cognate canonical miRNA. (B) Examples of miRNAs whose canonical isoform and isomiRs display distinct dynamics across development. Dynamics of the canonical miRNA (solid red line) and the top six most abundant isomiRs (dashed lines) are shown. 5’/3’ notation represents the miRNA terminus at which an alteration was identified; t = truncation; e = extension; final numerical value denotes the number of nucleotides added or lost. For example, ‘3′e1’ means that there was a single nucleotide extension of the 3’ end of the canonical miRNA. For remaining miRNAs’ dynamics, see Figure S3.

To determine if the isomiR expression across development generally followed the expression pattern of their canonical miRNAs, we identified and plotted abundance for each canonical miRNA and its top 6 isomiRs across *C. elegans* development (Fig. 7 and Supplemental Figures 4–6). We found that for some miRNAs, such as miR-58a-3p, miR-63-3p, miR-240-3p, miR-246-3p, miR-1022-5p, and miR-1829a-3p, isomiRs follow the same temporal expression pattern across *C. elegans* developmental stages as their canonical miRNA (Fig. 7(A)). Interestingly, for other miRNAs, such as miR-50-5p, miR-71-5p, miR-84-5p, miR-229-3p, miR-250-3p, and miR-791-3p, some isomiRs exhibited temporal dynamics that were distinct from their cognate canonical miRNA (Fig. 7(B)). *mir-71* has been previously shown to produce two dominant, age-dependent isoforms of miR-71-5p that exhibit unique dynamics in ageing *C. elegans* adults [62]. Our analysis confirmed the presence of the two most abundant miR-71-5p isoforms (canonical and miR-71-5p-3′t4) (Fig. 7(B) and 61), demonstrating their distinct dynamics across *C. elegans* developmental stages. While canonical miR-50-5p and its isomiR expression largely tracked across development, canonical miR-50-5p expression was highest in embryos, while a 2-nt truncated isoform peaked in L2/L3 and young adults (Fig. 7(B)). Surprisingly, we found *let-7* family member, *mir-84-5p*, to have high isomiR levels, especially at the L4 stage (Fig. 7(B)). miRtrons are generated independent of Drosha processing, but are still subject to Dicer processing, nuclease trimming (if tailed), and potential variations in splicing. We observed the presence of isomiRs for miRtrons [63,64] detected in our data (Supplemental Figures 4–6), suggesting that miRtron loci are capable of producing miRNA isoform heterogeneity.

**Figure 8.**
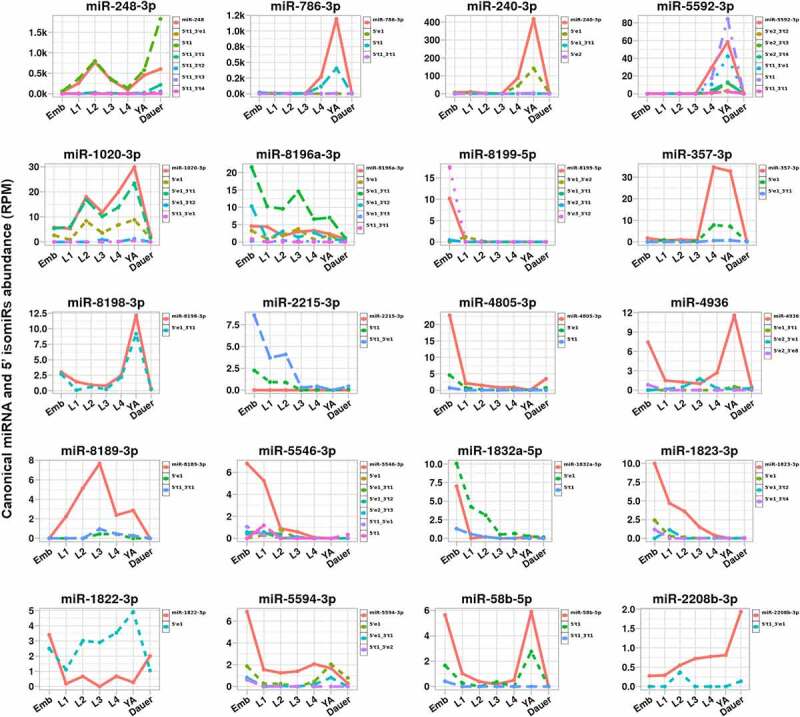
**Dynamics of seed-altering 5’ isomiRs**. For some miRNAs, isomiRs with alternative 5’ ends can be nearly as abundant as their cognate canonical miRNAs at some stages or represent a significant proportion (>10%) of the canonical miRNA reads. 3’ terminus-only isomiRs are not shown to highlight isomiRs that exhibit seed-altering changes at the 5’ end. Additional miRNAs with a significant proportion of 5’ isomiRs are plotted in Figure S4.

miRNA 5’ end truncations or additions result in the change of miRNA seed sequence and are predicted to alter its pool of miRNA targets. While 5’ isomiRs as a group did not represent a large proportion of miRNA loci-mapped reads (Fig. 5), we wanted to examine the extent to which 5’ isomiRs may be prevalent for individual miRNAs. To do so, we plotted abundances of canonical miRNAs and their 5’ end-altering isomiRs (Fig. 8 and Supplemental Figure S7). We found that in some cases, 5’ end isomiRs were just as abundant as their canonical miRNAs (Fig. 8). For example, a 5’ miR-248 isomiR, miR-248-5′t1, was more, or nearly as, abundant as the canonical miR-248 (Fig. 8). Same pattern was observed for isomiRs of miR-5592-3p, miR-1020-3p, and others (Fig. 8). In other cases, 5’ end isomiRs were less abundant than their canonical miRNAs, but still represented a substantial fraction (≥10%) of the canonical miRNA reads (Fig. 8 and Supplemental Figure S7). For example, miR-786-3p-5′t1 was one-third as abundant as canonical miR-786-3p, with miR-240-3p-5′e1 representing a similar fraction of the canonical miR-240-3p miRNA reads (Fig. 8).

### 5’ isomiRs are predicted to target larger, partially overlapping sets of target genes when compared with predicted canonical miRNA target pools

**Figure 9.**
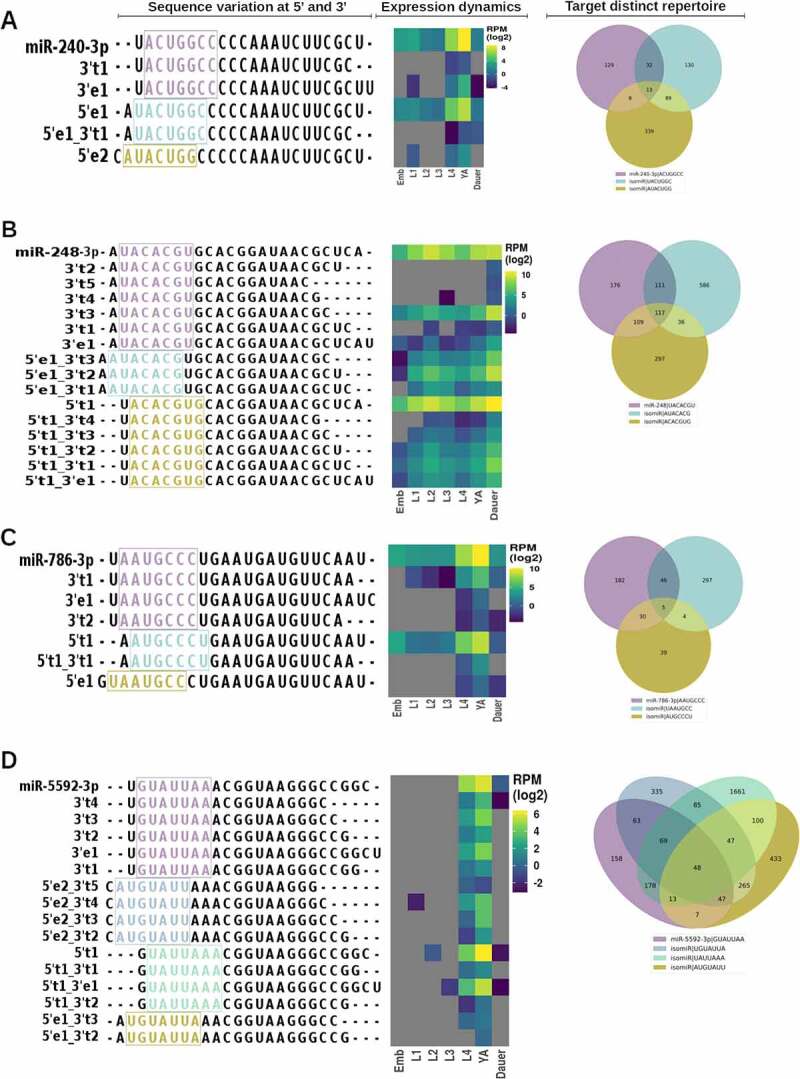
**Expression of miRNAs and 5’ isomiRs across stages and their predicted targets**. (A-D) miRNAs with a significant presence of 5’ isomiRs. IsomiR sequences and their expression vary across stages, with predicted target repertoires changing among canonical and altered seeds for miR-240 (A), miR-248 (B), miR-786 (C), and miR-5592 (D).

Since 5’ isomiR seed sequence differs from its canonical miRNAs’ seed, we assessed the extent to which such seed shifts may alter gene target pools. Using Targetscan [53] to predict miRNA targets, we found that 5’ isomiRs are predicted to target large, somewhat overlapping, but generally distinct groups of genes (Fig. 9). For example, while canonical miR-240-3p miRNA and its 3’ isomiRs are predicted to target 182 genes, a largely non-overlapping set of 264 genes may be targeted by its relatively abundant 5’ isomiR, miR-240-3p-5′e1 (Fig. 9(A)). Similarly, highly expressed miR-248-3p isomiRs with a 5’ end 1-nt truncation are predicted to target a substantial, largely non-overlapping set of genes compared to the canonical mir-248-3p and its non-seed changing isomiRs (Fig. 9(B)). Abundant 5’ isomiRs of miR-786-3p (Fig. 9(C)) and miR-5592-3p (Fig. 9(D)) are similarly predicted to target mostly non-overlapping sets of genes. As this analysis relies on target predictions, it remains to be seen to what extent 5’ isomiRs target genes for post-transcription repression. However, these small changes in miRNA sequence have the capability to dramatically expand the miRNA gene-targeting repertoire.

While 3’ isomiRs are not predicted to affect seed-based gene targeting, supplemental 3’ end targeting has been shown to play a role in target identification [58,59]. In some cases, a single nucleotide change at the 3’ end of miRNAs can expand or diversify the target repertoire for a given miRNA locus by increasing 3’ miRNA end complementarity to target mRNAs [31,59]. To broadly assess the potential effects of isomiRs on gene targeting throughout development, we plotted Z-scores of canonical miRNAs and isomiR abundance across stages to create stage-based clustering (Supplemental Figure S8). Canonical miRNAs (Supplemental Figure S8A) showed a clustering pattern that was distinct from their collective isomiRs (Supplemental Figure S8B), suggesting that, as a significant subclass of miRNAs reads, 3’ and 5’ isomiRs could potentially affect the transcriptional landscape during *C. elegans* development.

## Discussion

miRNAs are potent post-transcriptional regulators of gene expression. Small RNA sequencing is frequently used in conjunction with other experiments to study the prevalence, dynamics, and function of miRNAs in development and disease. Typical small RNA sequencing analysis aggregates all reads mapping to a single miRNA locus to determine the abundance of miRNAs produced from that locus. However, miRNA populations are not homogeneous, and reads that map to the same locus may harbour changes that significantly alter miRNAs targeting capability. Variations in library preparation, sequencing methods, and data analysis can introduce discrepancies and complicate identification of exact miRNA sequences and their isoforms. This work provides a detailed assessment of templated isomiR populations across *C. elegans* development to evaluate the potential contributions of isomiRs to gene regulation, using a consistent method of library preparation and data analysis. Since miRNA isoforms can dramatically expand and/or diversify the target repertoire of miRNAs, our characterization of templated isomiR prevalence is an important first step at understanding the impact of these miRNA isoforms on gene expression landscape in development.

To determine the potential impact of templated alternative miRNA isoforms on gene regulatory landscape during *C. elegans* development, we sequenced small RNAs for each distinct developmental stage using a sequencing method designed to minimize biases introduced during library preparation and data processing. We found that most of the *C. elegans* miRNA loci do not produce a substantial fraction of templated isomiRs, with canonical miRNA sequences being the dominant, most abundant miRNA species (Fig. 3 and Supplemental Figure 1). In rare cases, the observed most dominant miRNA species in our data was an isomiR, suggesting a possibility of previous missanotations (Fig. 3 and Supplemental Figure 1). In other cases, isomiR prevalence changed across the developmental stages, raising an intriguing possibility that alternative DROSHA or DICER processing could be developmentally regulated (Fig. 3, Supplemental Figure 2, Fig. 7, and Supplemental Figures 4–6). Overall, the high amount of isomiRs generated for some miRNA loci suggests that these miRNAs may undergo relatively frequent alternative cleavage events. It will be interesting to determine, in future studies, whether primary and precursor miRNA molecules share sequence and/or structure features that make them more prone or more resistant to alternative cleavages. It is important to note, however, that truncated miRNA isoforms could represent degradation products of full-length miRNAs, generated through trimming mechanisms rather than alternative enzymatic processing.

As a subclass of isomiRs, seed-changing 5’ isomiRs represented a small fraction of the global miRNA-mapped reads. However, several miRNA loci produced abundant 5’ isomiRs, which were as abundant as canonical miRNA reads or represented a significant proportion of canonical miRNA reads (Fig. 8). These 5’ isomiRs potentially expand the miRNA targeting repertoire (Fig. 9). It is tempting to speculate that these isomiRs have biologically relevant targeting functions distinct from their canonical miRNAs. However, the extent to which the identified *C. elegans* 5’ isomiRs represent functioning miRNA isoforms remains to be determined and will need to be experimentally validated in the future. Previous studies in other systems have shown Argonaute loading of isomiRs [19–21], demonstrating the functional importance of isomiRs [19,37]. Sequencing experiments linking miRNA molecules to their targets [65,66], performed with sufficient depth, can in the future provide and expand information on isomiR targeting abilities.

Our analysis *of C. elegans* isomiRs focused on characterizations of templated isomiRs only. Untemplated isomiRs can include 3’ end modification such as uridylation and adenylation as well as internal miRNA sequence alternations such as A-to-I or C-to-U editing [2,24]. While 3’ end poly-uridylation has been shown to block miRNA biogenesis and target the pre-miRNAs for degradation [67,68], mono-uridylation and mono-adenylation of 3’ miRNA generally have a positive effect on miRNA processing and stability [28,69–71]. 3’ mono-uridylation promotes Dicer processing and loss of uridylases can lead to a decrease in abundance for miRNAs dependent on mono-urydilation for processing [70]. Similarly, mono-urydilation has been recently shown to alter miRNA strand selection through alternative Dicer processing [42]. As such, current [72] and future studies into *C. elegans* untemplated isomiRs will continue to expand the isomiR landscape and the roles of 3’ end modifications in *C. elegans* development.

Since many miRNAs have evolved through duplication and diversification [73,75], it is tempting to speculate that alternative processing of newly duplicated miRNA loci could contribute to evolution of new miRNAs. Establishment of a previously inabundant 5’ end isomiR as a new dominant miRNA isoform with a new seed could expose new sets of genes to repressive miRNA regulation. The extent to which templated isomiRs, generated through alternative processing, have contributed to miRNA evolution will no doubt come to light as isomiR populations are more closely examined and characterized across the evolutionary tree.

## Supplementary Material

Supplemental Material

## Data Availability

The data supporting the findings of this study are available within the article and its supplemental materials. Sequencing data files are available on NCBI Sequence Read Archive (SRA) under the accession number SUB11409514 with bioproject id: PRJNA834082. https://www.ncbi.nlm.nih.gov/bioproject/PRJNA834082
