# Supplementary material for: Global profiling and annotation of templated isomiRs dynamics across Caenorhabditis elegans development
Source: RNA Biol. 2022 Jul 18;19(1):928–42. doi: 10.1080/15476286.2022.2099646 (PMC9298154; doi:10.1080/15476286.2022.2099646)
Supplement: Supplemental Material [file KRNB_A_2099646_SM3242.zip › Supplemental_Figure_2.pdf]

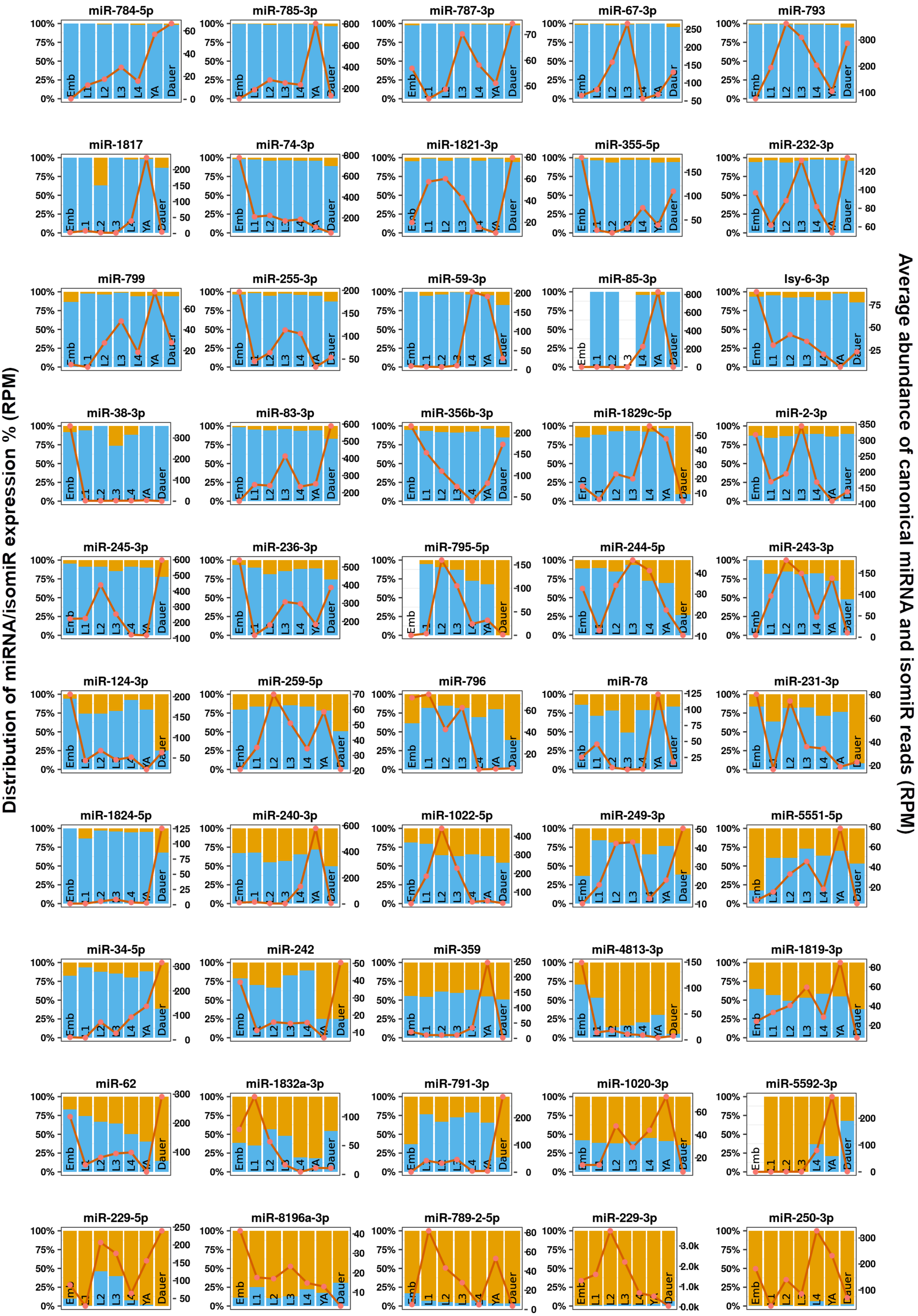

Supplemental Figure 2.
