# Supplementary material for: Global profiling and annotation of templated isomiRs dynamics across Caenorhabditis elegans development
Source: RNA Biol. 2022 Jul 18;19(1):928–42. doi: 10.1080/15476286.2022.2099646 (PMC9298154; doi:10.1080/15476286.2022.2099646)
Supplement: Supplemental Material [file KRNB_A_2099646_SM3242.zip › Supplemental_Table_1.pdf]

Supplemental Table 1. Small RNA-seq libraries from seven stages, including Embryo, L1-L4, Young adult, and dauer were utilized for isomiR identification. For each library, the read statistics following each step of the pipeline (see Figure 2) are provided. Genome mapped, filtered reads were used for isomiR identification.

Samples	Raw reads	Total filtered reads	Filtered collapsed reads (unique)	Reference genome alignment: Total mapped reads	Reference genome alignment: Collapsed reads (unique)	Extracted 4S?4S Cigar flag**: Total reads	Extracted 4S?4S Cigar flag**: Collapsed reads (unique)
N2_Emb_rep1	17,644,973	16,026,575	1,600,631	14,069,465	610,761	4,822,578	309,071
N2_Emb_rep2	18,542,496	17,457,874	1,518,053	11,431,169	635,049	5,939,024	316,747
N2_Emb_rep3	16,829,287	15,929,593	2,258,869	15,932,890	1,000,979	5,456,492	502,248
N2_Emb_rep4	8,235,464	5,668,806	2,796,372	4,716,876	962,004	1,413,048	565,475
N2_L1_rep1	14,008,871	13,103,304	2,131,431	8,948,622	960,078	4,289,458	459,395
N2_L1_rep2	18,572,446	17,264,265	1,431,606	11,127,724	568,768	5,680,647	263,321
N2_L1_rep3	19,939,433	18,142,134	1,356,323	15,562,364	557,584	5,966,050	264,122
N2_L2_rep1	22,105,367	20,814,601	2,339,656	13,803,539	1,010,908	6,908,528	480,125
N2_L2_rep2	15,934,240	14,617,311	1,464,748	9,066,733	651,262	4,365,345	309,444
N2_L2_rep3	15,569,805	14,540,616	2,601,851	9,583,943	1,232,299	4,326,847	583,453
N2_L3_rep1	21,640,050	20,495,895	1,517,838	12,963,375	620,410	6,727,537	288,886
N2_L3_rep2	15,296,069	14,244,630	1,934,255	9,536,705	887,865	4,705,297	422,069
N2_L3_rep3	26,655,383	25,328,132	2,541,931	16,386,047	1,118,662	8,219,493	513,660
N2_L4_rep1*	18,740,945	17,267,301	3,428,964	12,185,301	1,650,744	5,832,988	805,386
N2_L4_rep2*	17,757,542	15,823,738	2,896,986	11,017,241	1,379,635	5,166,244	652,786
N2_L4_rep3*	22,690,120	21,353,668	3,702,131	15,060,916	1,794,903	7,452,707	864,804
N2_YA_rep1	18,428,037	17,540,596	1,353,321	11,482,803	556,809	5,438,359	278,613
N2_YA_rep2	13,773,178	12,966,213	3,443,819	9,088,576	1,693,204	3,965,256	850,929
N2_YA_rep3	13,908,496	12,658,216	3,432,629	8,890,513	1,614,176	3,879,998	817,127
N2_Dauer_rep1	21,747,053	20,465,931	2,198,577	14,953,682	936,317	6,692,036	427,838
N2_Dauer_rep2	17,060,590	15,845,394	1,885,259	10,751,282	859,511	5,119,734	377,573
N2_Dauer_rep3	18,518,741	17,420,542	2,107,058	11,790,564	919,148	5,698,427	423,081

*Samples were taken from Li et al, 2019
**Reads were extracted from genome alignment with 4 nt randomer included at 5’ and 3’ ends
