# Supplementary figures and images for: Global profiling and annotation of templated isomiRs dynamics across Caenorhabditis elegans development
Source: RNA Biol. 2022 Jul 18;19(1):928–42. doi: 10.1080/15476286.2022.2099646 (PMC9298154; doi:10.1080/15476286.2022.2099646)

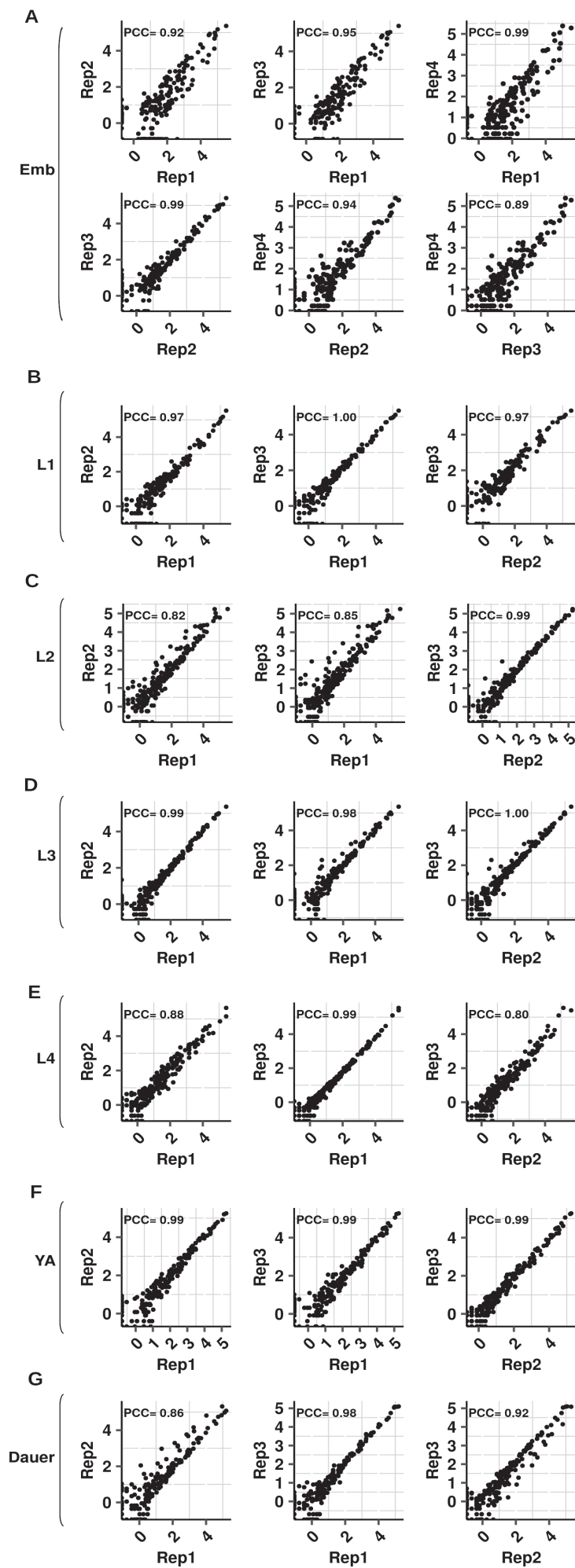

Supplemental Figure 1.

Supplement: Supplemental Material [file KRNB_A_2099646_SM3242.zip › Supplemental_Figure_1.pdf]

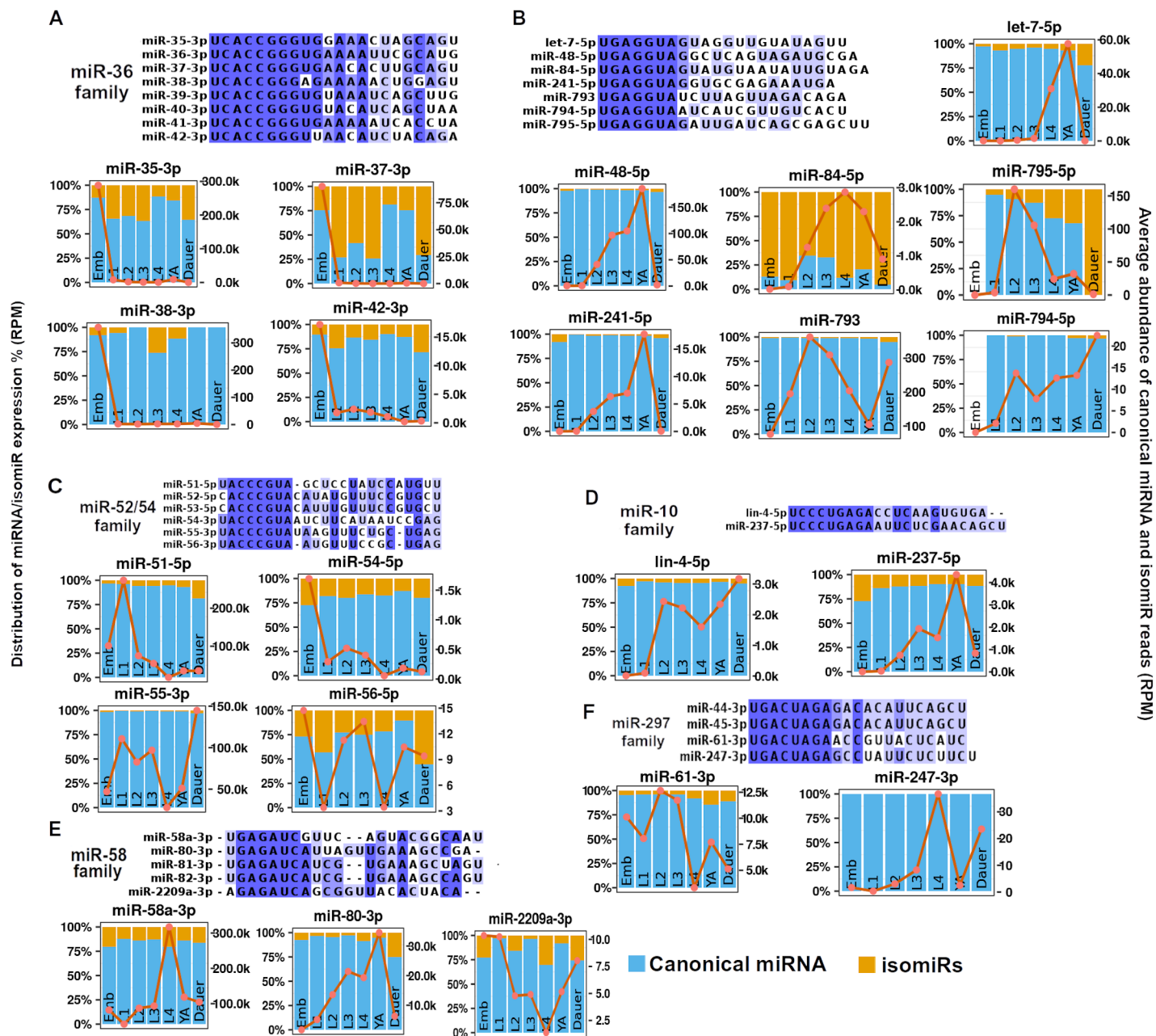

Supplemental Figure 3.

Supplement: Supplemental Material [file KRNB_A_2099646_SM3242.zip › Supplemental_Figure_3.pdf]

# Canonical miRNA and isomiRs abundance (RPM)

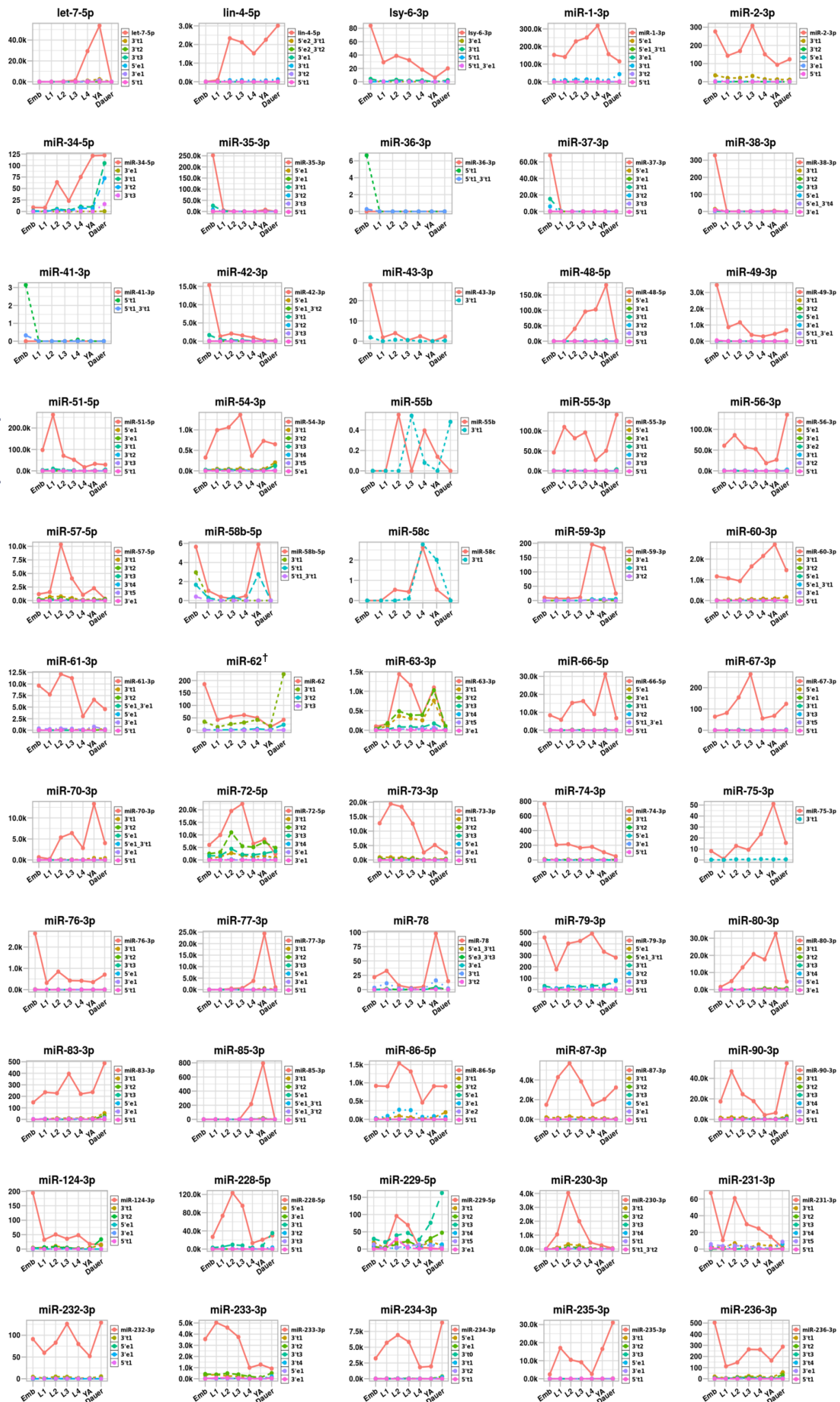

Supplemental Figure 4.

Supplement: Supplemental Material [file KRNB_A_2099646_SM3242.zip › Supplemental_Figure_4_revised.pdf]

Canonical miRNA and isomiRs abundance (RPM)

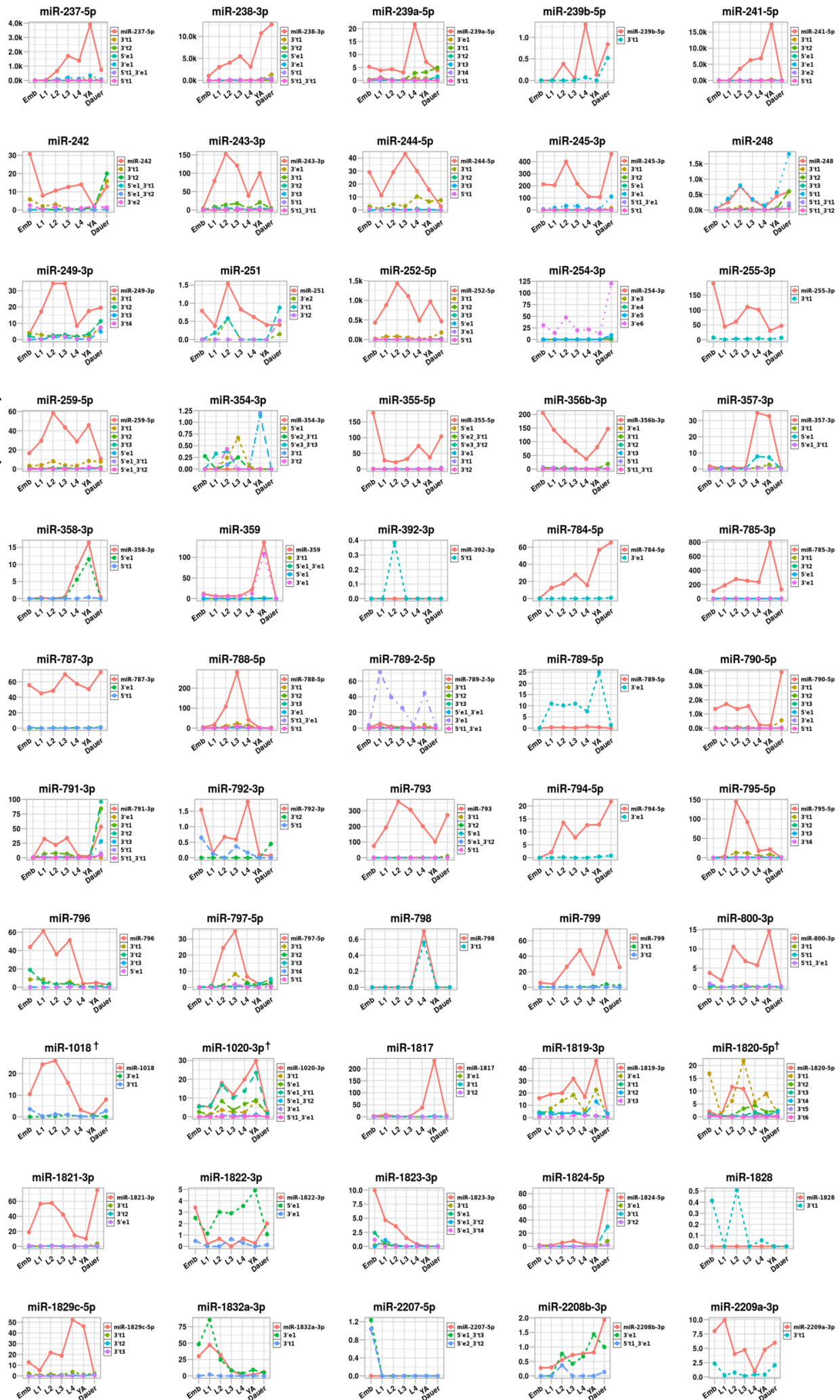

Supplemental Figure 5.

Supplement: Supplemental Material [file KRNB_A_2099646_SM3242.zip › Supplemental_Figure_5_revised.pdf]

Canonical miRNA and isomiRs abundance (RPM)

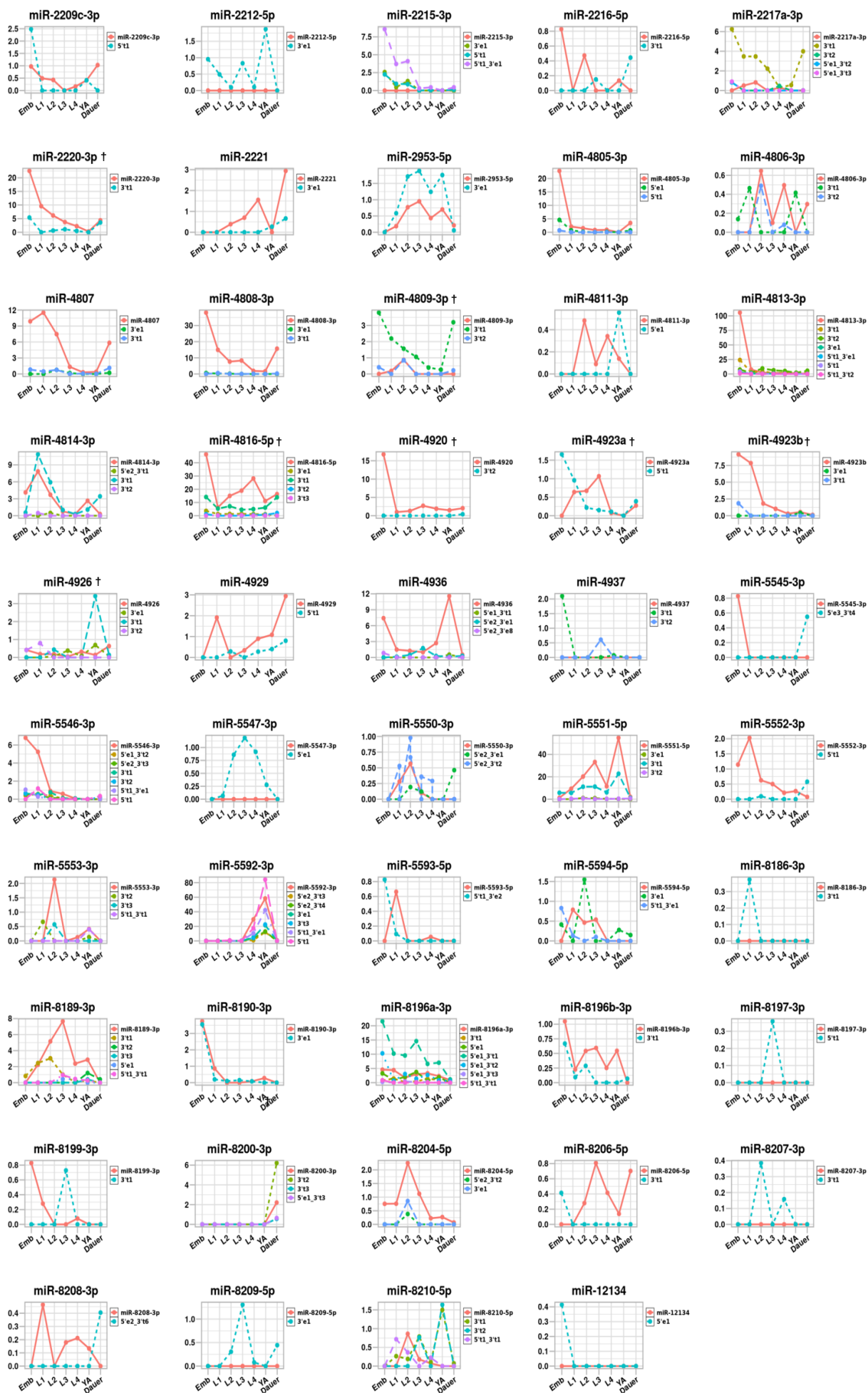

Supplemental Figure 6.

Supplement: Supplemental Material [file KRNB_A_2099646_SM3242.zip › Supplemental_Figure_6_revised.pdf]

Canonical miRNA and 5' isomiRs abundance (RPM)

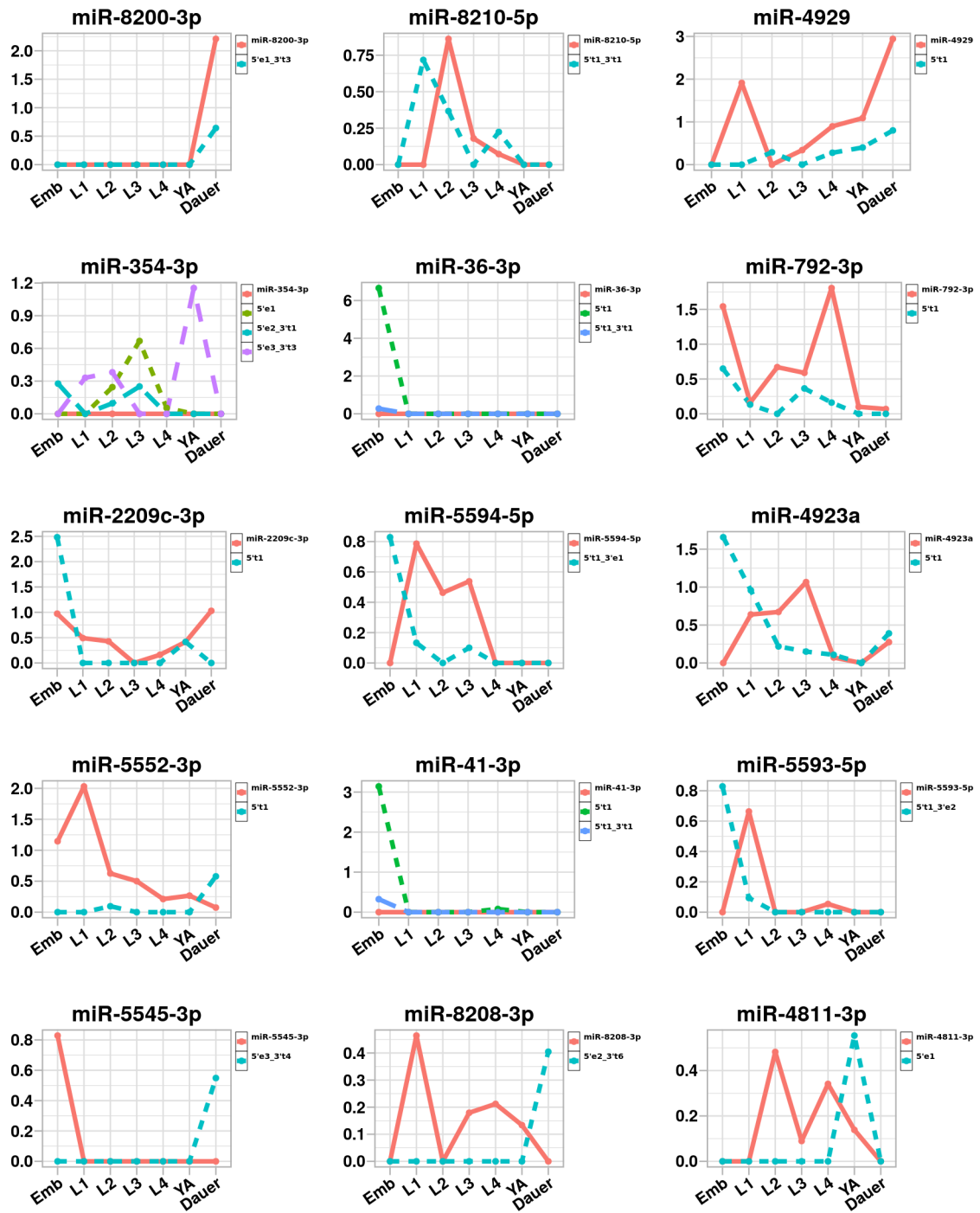

Supplemental Figure 7.

Supplement: Supplemental Material [file KRNB_A_2099646_SM3242.zip › Supplemental_Figure_7.pdf]

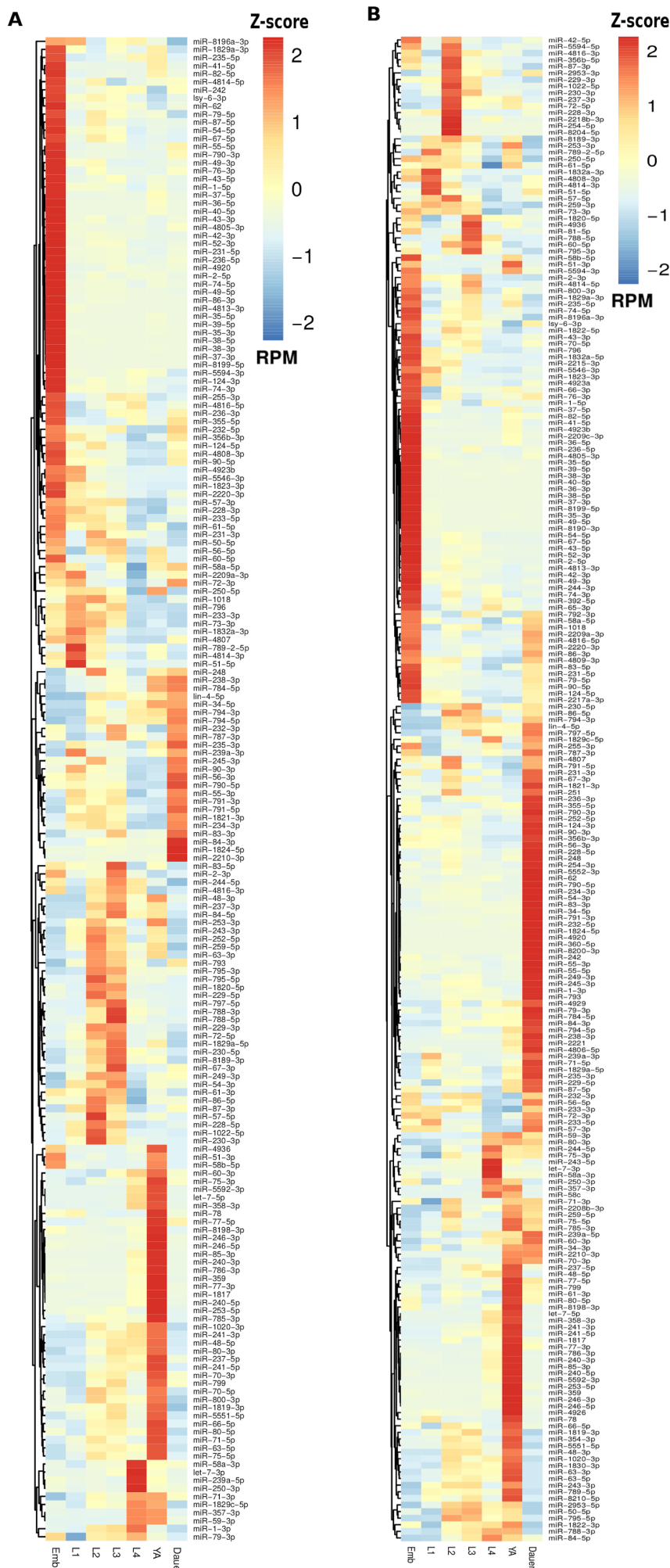

Supplemental Figure 8.

Supplement: Supplemental Material [file KRNB_A_2099646_SM3242.zip › Supplemental_Figure_8.pdf]
